# Sociability

**Published:** 1876-06

**Authors:** 


### SOCIABILITY.

We once heard a professional gentleman, at his table, say, that he "never did a stroke of work in his life." We looked about his house, and came to the conclusion that his wife and servants could pretty near say the same thing. On leaving him, we saw the well, hard by; and the sum total of conveniences for getting water for the supply of the whole family was—a rope, one end of which was tied to a tree, the other to a tin pan. A lady once told us, at a dinner-table, with apparent pride, that she had not a poor blood-relation in the world, until the last generation, which found them and herself in proximity to the little end of nothing—sharpened. Let your guests see and feel, that in all you do, or think, or say, there is truth, earnestness, goodwill, and courtesy. Let the aim be not merely to spread the table with luxuries, with things early and rare, but let the surroundings be abundant in smiles, in kind-heartedness, in high-bred courtesies, in genial humor, in conversation on subjects not only pleasurable in themselves, but in their suggestions, and let everything "go off" with that unselfish *abandon*, with that careless goodnature, that decorous unreserve, which throw around both guest and host the delightful atmosphere of home.

If people in the city could thus visit one another, and allow their "country" cousins and acquaintances to visit them, an incalculable benefit would redound to the physical man; most especially would it have a healthful effect on the minds and bodies, and tempers of our wives and daughters, by that change of food, and air, and subject of thought, and feeling, and conversation, which is essentially necessary to our well-being. Were dinner and tea thus taken from home, two or three times a week, by every woman in New York, old and young, it would not only add to the bodily and mental health of the individual, but society would be benefited by that warming up of our sympathies, our humanities, and that general kindliness of heart which such associations engender, and which so elevate our nature. We are social beings: "man was not made to be alone." There is a yearning in all hearts for company, for congenial associates; and by its wise indulgence, man promotes manliness, and women cultivate those charms of character which make them on earth our guardian angels.

